# Green synthesis of silver doped zinc oxide/magnesium oxide nanocomposite for waste water treatment and examination of their cytotoxicity properties

**DOI:** 10.1016/j.heliyon.2024.e30374

**Published:** 2024-04-25

**Authors:** Toktam Shekofteh Narm, Habib Hamidinezhad, Zahra Sabouri, Majid Darroudi

**Affiliations:** a Department of Physics, Faculty of Basic Sciences, University of Mazandaran, Babolsar, Iran; b Nanobiotechnology Research Group, University of Mazandaran, Babolsar, Iran; c Student Research Committee, Mashhad University of Medical Sciences, Mashhad, Iran; d Nuclear Medicine Research Center, Mashhad University of Medical Sciences, Mashhad, Iran; e Department of Medical Biotechnology and Nanotechnology, Faculty of Medicine, Mashhad University of Medical Sciences, Mashhad, Iran

**Keywords:** Green synthesis, Silver-doped zinc oxide/magnesium oxide nanocomposite, Photocatalytic, Cytotoxicity

## Abstract

This research attempted to prepare silver-doped zinc oxide/magnesium oxide nanocomposite (Ag-doped ZnO/MgO-NCP) using *Mentha pulegium* plant extract. The synthesized NCP was investigated by X-ray diffraction analysis (XRD), Fourier Transform Infrared (FT-IR), Field Emission Scanning Electron Microscope (FESEM), Energy dispersive X-ray spectroscopy (EDX), Mapping, and UV–Visible analyses. The XRD data displayed cubic crystal structures for silver & magnesium oxide and a hexagonal framework for zinc oxide. Also, FESEM and PSA images of NCP pointed out, that the average size of the spherical morphology is about 10–16 nm, while the analysis of EDX confirmed the attendance of Zn, Mg, Ag, and O elements. Under UVA light, we tested the photocatalytic activity of NCP to the degradation of Methylene blue (MB) and Rhodamine B (RhB) dyes in various temperatures (400, 500, and 600 °C). The results of the photocatalytic test displayed that the degradation percentage of MB dye in pH = 9, nanocomposite amount ∼30 mg, and dye concentration ∼1 × 10 ^−5^ M was about 98 %. We also evaluated the cytotoxicity of nanocomposite on cancer CT-26 cell line through the MTT method and obtained an IC_50_ value of 250 μg/mL.

## Highlights

• An eco-friendly and cost-effective procedure for the synthesis of Silver-doped Zinc Oxide/Magnesium Oxide Nanocomposite.
• Silver-doped Zinc Oxide/Magnesium Oxide Nanocomposite as an excellent photocatalyst for the degradation of organic dyes.
• Low cell bio-toxicity of the Silver-doped Zinc Oxide/Magnesium Oxide Nanocomposite.
• Application of *Mentha pulgium* extract for the biosynthesis of Silver-doped Zinc Oxide/Magnesium Oxide Nanocomposite.

## Graphical abstract

**Figure:**
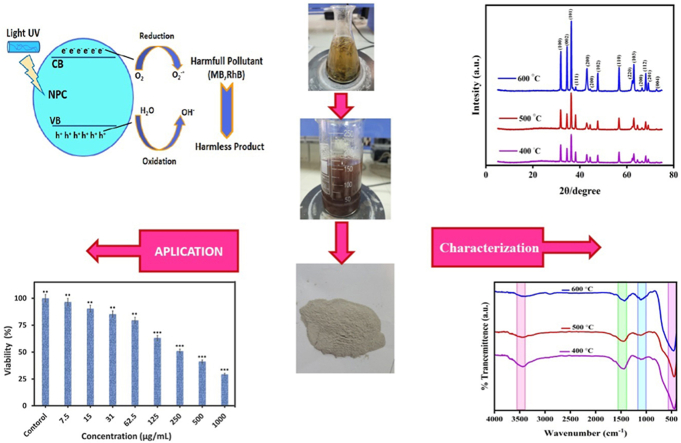

## 1 Introduction

The excessive speed of industrial developments in various fields, including textile, plastic, and paper, is an environmental challenge that is associated with the generation of great amounts of wastewater consisting of synthetic dyes [1,2]. Since artificial colors can have a negative impact on both the aquatic environment [3] and human health [4,5]. The environmentally friendly process of preparing nanomaterials by green synthesizing techniques as a catalyst for destroying the organic dyes of wastewater has been considered an alternative to chemical methods. Metal oxide nanoparticles (MONP) and metal nanoparticles (MNP) can offer special features for many applications such as catalysts [6,7]. ZnO is an instance of a well-known catalyst due to its stability and high oxidation power, which can consequently cause destructive effects on diverse organic dye effluents such as methylene MB and RhB [8,9]. The varying environmental utilization of semiconductor photocatalysts includes the mineralization of organic pollutants, water disinfection, etc. [10]. There are reports on the increasing performance rate of ZnO in the formation of composites with other metal oxides, which is known as an n-type semiconductor with an energy band gap of about 3.37 eV [11,12]. Moreover, magnesium oxide contains various characteristics such as high chemical behavior and photostability, low dielectric constant, and large band gap values that have led to its many applications in different areas including catalysis, antimicrobial, antioxidant, anticancer, and supercapacitor [[13], [14], [15]]. Different methods of synthesizing nanocomposites exist, including sol-gel, hydrothermal, laser ablation, co-precipitation, and sonochemical [16]. The electronic coupling between ZnO and MgO has improving effects on the optical attributes of ZnO–MgO nanocomposite and also increases the band gap energy [17]. The doping of ZnO nanocomposites with cobalt, copper, nickel, and silver, can extend their optical, electrical, and biological properties [[18], [19], [20], [21]]. The usefulness of stable silver nanoparticles, manufactured by green methods, is caused by their simplicity, low cost, and environmental friendliness features [[22], [23], [24]]. Despite the popularity of physical and chemical procedures as the most common preparation methods for nanocomposites, they proved to be expensive and dangerous to the environment. Lately, the interest of many has been captivated by the green synthesis of nanocomposites using plants due to the involvement of bioactive combinations, accessibility, and low cost [25,26]. The flowering aerial parts of the *Mentha pulgium* plant contain antiseptic attributes and have been traditionally used for the treatment of cholera, cold, food poisoning, bronchitis, and tuberculosis, as well as applied in the forms of expectorant, antitussive, carminative, menstruate, and diuretic [27]. According to recent studies, the effect of enhancing several middle metals on the properties of photocatalysts has been examined. V. Jagadeeswar et al. produced silver-doped zinc oxide (Ag/ZnO), which caused enhanced anticancer and photocatalytic capabilities [28]. The target of this research was to execute the synthesis of NCP by *Mentha pulegium* plant extract. After confirmation and characterization, we evaluated the photocatalytic acting of NCP in the degradation of RhB and MB dyes under UV-A light, which was followed by evaluating its cytotoxicity on cancer CT-26 cells through an MTT method.

## 2 Experimental

### 2.1 Materials and method

Fresh *Mentha pulegium* plant was collected from Narm village in South Khorasan, Iran. Magnesium nitrate hexahydrate (Mg (NO_3_)_2_·6H_2_O), Zinc nitrate hexahydrate (Zn (NO_3_)_2_·6H_2_O), Silver nitrate (Ag (NO_3_)_2_), Methylene blue (C_16_H_18_ClN_3_S), Rhodamine B (C_28_H_31_ClN_2_O_3_), and Sodium hydroxide (NaOH) were purchased from Merck and Sigma Companies.

### 2.2 Preparation of *Mentha pulegium* plant extract

To prepare *Mentha pulegium* plant extract, 2.0 g of *Mentha pulegium* plant was added to 100 mL water solvent to be stirred at 55 ^°^C for 2 h. The obtained extraction was filtered, and stored in a refrigerator.

### 2.3 Synthesis of Ag-doped ZnO/MgO-NCP

**Fig. 1:**
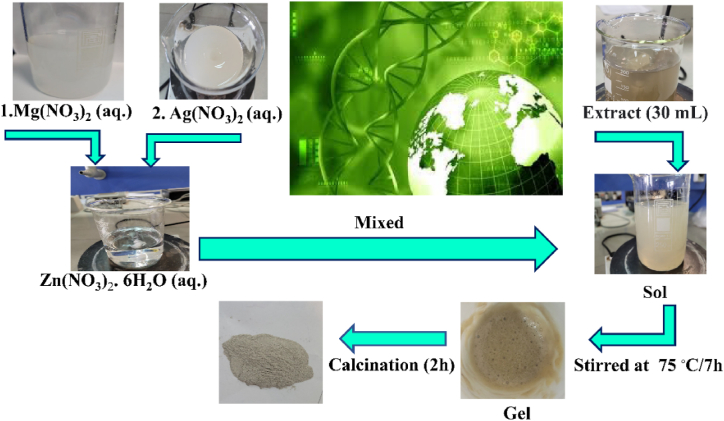
The schematic biosynthesis of nanocomposite.

To prepare NCP, 7.43 g of Zn (NO_3_)_3_.6H_2_O, 6.41 g of Mg (NO_3_)_3_.6H_2_O and 0.042 g of Ag (NO_3_)_3_ salts were separately dissolved in 50 mL of distilled water and stirred at 24 °C for 20 min. Then, magnesium nitrate and silver nitrate solutions were slowly added to the zinc nitrate solution and stirred at ambient temperature. In the following, 30 mL of *Mentha pulegium* plant extract was slowly added to the mixed solution to have the resultant sol stirred at 75 °C for 7 h. The produced gel underwent calcination at various temperatures (400, 500, and 600 °C) for 2 h to prepare NCP. The schematic biosynthesis of nanocomposite is shown in Fig. 1.

### 2.4 Characterization

The surface attributes of the synthesized nanocomposite, including phase, shape, and size, were investigated through the outcomes of XRD, FESEM, EDX, PSA, and Mapping analyses. FTIR analysis and optical detection UV–Vis spectrophotometry were exerted to determine the functional groups. Moreover, next to investigating the photocatalytic functionality for the degradation of MB and RhB dyes and also the cytotoxic effects evaluated against CT-26 cancer cells.

### 2.5 Photocatalytic

The synthesized Ag-doped ZnO/MgO-NCP at various temperatures (400, 500, and 600 °C) was investigated for the photocatalytic process for the photodegradation of MB and RhB pigments under UV light. for this purpose, we used a semi-manual reactor to disperse the synthesized samples in the organic pollutant solution (MB and RhB). First, 0.01 g of the nanocomposite was dispersed in MB and RhB solutions (100 mL of 1 × 10^−5^ M) to be stirred for 45 min in a dark room. Then, the solution was placed under UV light to remove and centrifuge 2 mL of it (12,000 rpm, 10 min) every 20 min. The absorption of solutions at different intervals (20 min) was read by the wavelengths of UV–Vis spectrophotometer at 555 nm (RhB) and 663 nm (MB). The percentage of dye degradation was determined according to Eq. (1) [29].

(1) $$\text{Degradation}\;\left(\%\right)=\frac{{A_{o}-A}_{t}}{A_{o}}\times 100$$

where A_0_ represents the initial absorption are UV radiation and A_t_ refers to the absorption of solution at time t.

### 2.6 Cytotoxicity

#### 2.6.1 Cell culture

In this section, the cytotoxicity of nanocomposite was surveyed on a CT-26 cancer cell line (murine colorectal carcinoma), which was prepared from Tehran, Pasteur Institute, Iran. The cells were incubated in DMEM media enriched with fetal bovine serum (FBS, 10 %) and antibiotics (1 %) in conditions 37 °C, CO_2_ (5 %), and humidity (95 %).

#### 2.6.2 MTT assay

The toxicity of this nanocomposite was evaluated on the CT-26 cell line by MTT test. The process was initiated by incubating the cells (100 μL, 1 × 10^4^ per well) per well of the 96-well plate, Then cells were exposed to different concentrations of nanocomposite (0, 125, 250, 500, and 1000 μg/mL) for 24 h. Afterward, the MTT (25 μL, 5 mg/mL) was added to each well to perform incubation for 4 h [30], during which MTT was deoxidized with succinate dehydrogenase which is one of the enzymes of the mitochondrial respiratory cycle. The regeneration and separation of this ring procreate the brilliant blue-violet crystals of formazan. To dissolve the formazan crystals, 100 μL of DMSO was added to each well and shaken for about 15 min. Lastly, the absorbance was read at 570 nm and the Cell survival percentage was determined based on Eq. (2) [31].

(2) $$Viability\;\left(\%\right)=\frac{A_{t}}{A_{c}}\times 100$$

Where A_t_ shows the absorbance of the sample, A_c_ refers to the absorbance of the control.

## 3 Result and discussion

### 3.1 UV–Vis

**Fig. 2:**
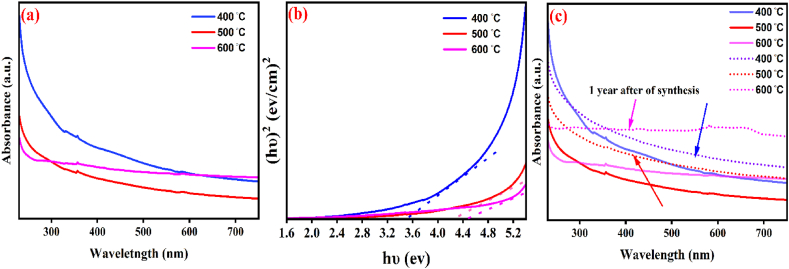
The spectra of UV–Vis (a), Bandgap (b), and Stability (c) of NCP.

The optical properties of nanocomposite were evaluated by UV–Vis spectrophotometry about 200–800 nm. The absorption bands of synthesized Ag-doped ZnO/MgO-NCP at 400, 500, and 600 °C were displayed at 358 and 363 nm, respectively (Fig. 2(a)). We also determined the bandgap energy of our product by Eq. (3), involving the plotting of (αhυ)^2^ vs. photon energy (hυ), and the energy axis's intercept is extrapolated [32].

(3) $${\left(\alpha hv\right)\;}^{n}=A\;\left(hv\;–\;Eg\right)$$

where α is the absorption factor, hv refers to the photon energy, Eg is the band gap energy, and n is equal to 2 or 0.5 for direct or indirect transmissions. The values of band gap energy at 400, 500, and 600 °C were about 3.58, 4.58, and 4.78 eV, respectively (Fig. 2 (b)). Also, the plot of catalyst stability is shown in Fig. 2 (c).

### 3.2 FTIR spectra

**Fig. 3:**
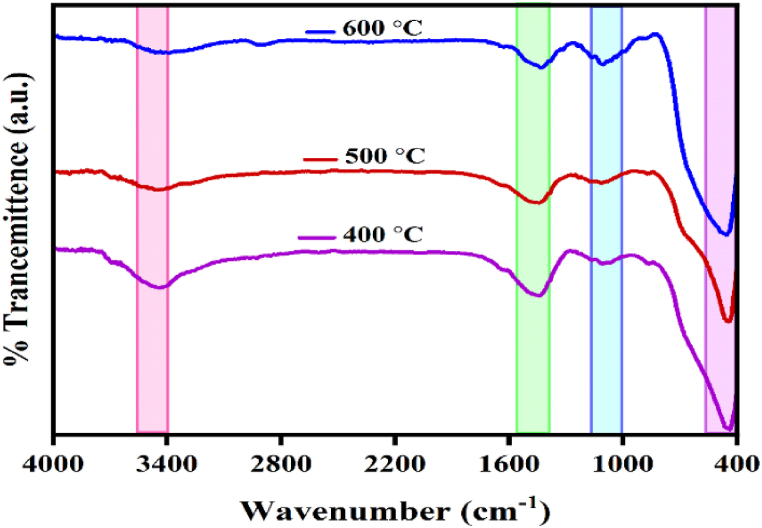
FTIR spectra of NCP.

Fig. 3 provides the FTIR spectra of our nanocomposite within the limit of 400–4000 cm^−1^. The peaks beneath 1000 cm^−1^ are attributed to the existence of metal and oxygen bonding vibrations [8]. The other strong peaks are about 462 cm^−1^ and 439 cm^−1^ which may be associated with the presence of Zn–O and Mg–O bands. In addition, the peaks at 1442 cm^−1^ were due to the vibration modes of residual nitrates, while the relatively wideband at 3439 cm^−1^ could be related to the O–H stretching vibrations of water molecules [30].

### 3.3 XRD pattern

**Fig. 4:**
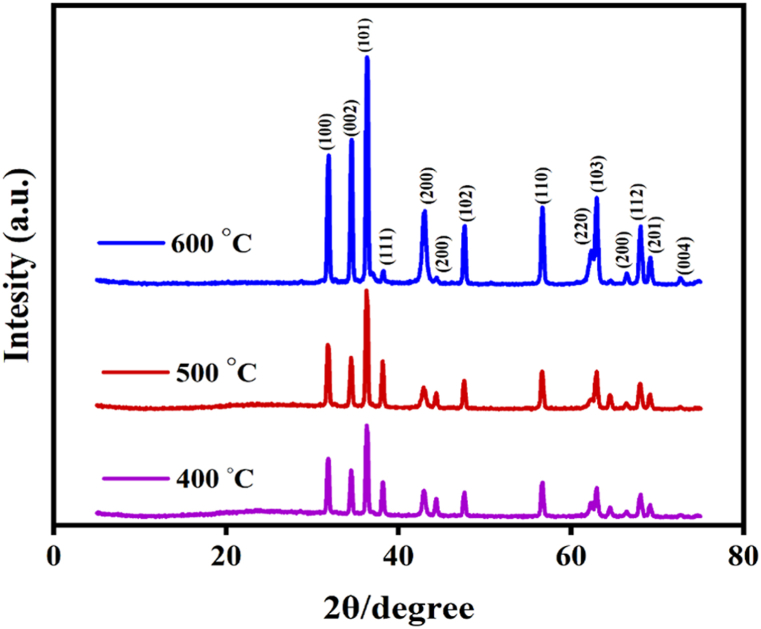
XRD pattern of NCP.

Fig. 4 exhibits the XRD pattern of the Ag-doped ZnO/MgO-NCP. According to this analysis, the crystal structure of zinc oxide is hexagonal and the peak positioning is formed at the angles of 31.8, 34.5, 36.3, 47.6, 56.6, 62.9, 67.9, 69.2, and 72.5° which match to the schemes of (100), (002), (101), (102), (110), (103), (200), (112), (201), and (004) in order, while the diffraction peaks conform with the standard samples (JCPDS file No. 36–1451) [33]. The crystalline structure of magnesium oxide is cubic and the peak location at the angles of 37.6, 42.9, and 62.2° matches the schemes of (111), (200), and (220) in order, while the diffraction peaks confirm by standard sample (JCPDS card No. 89–7746) [34]. Moreover, the crystalline structure of Ag is cubic and the peak location at the angles of 38, 44.08, and 64.40° matches the schemes of (111), (200), and (220) in order, as the diffraction peaks conform with the standard sample (JCPDS. No. 65–2871) [35]. The crystalline sizes **(grain size)** were determined through the Scherrer Deby formula Eq. (4) [36].

(4) $$D=\frac{K\lambda }{\beta \;\cos\;\theta }$$

D is the crystalline size in nanometers, λ refers to the wavelength of radiation (0.154 nm), constant k is equaled to 0.94, β is the peak width at half-maximum, and θ presents the peak positioning [37]. According to the results, the diverse crystal sizes of prepared nanocomposite calcined at different temperatures can increase the rate of peak intensity. The % crystallinity and specific surface area were computed using Eq. (5) and Eq. (6) respectively [[38], [39], [40], [41]]. The gathered outcomes were inserted in Table 1.

(5) $$\text{Crystallinity}\;\left(\%\right)=\frac{\text{crystalline}\;\text{spik}e^{\prime }s\;\text{area}}{\text{overall}\;\text{area}}\times 100$$

(6) $$\text{Surface}\;\text{area}=\frac{6000}{\;D\times \rho_{x}}$$

**Table 1:** Results of XRD pattern.

Temp (^◦^C)	Grain size (nm)	Crystallinity(%)	Surfacearea (m^2^)
400	27.3	62	154.79
500	27.8	65	152.01
600	28.2	83	149.85

### 3.4 FESEM/PSA/EDX/Mapping

**Fig. 5:**
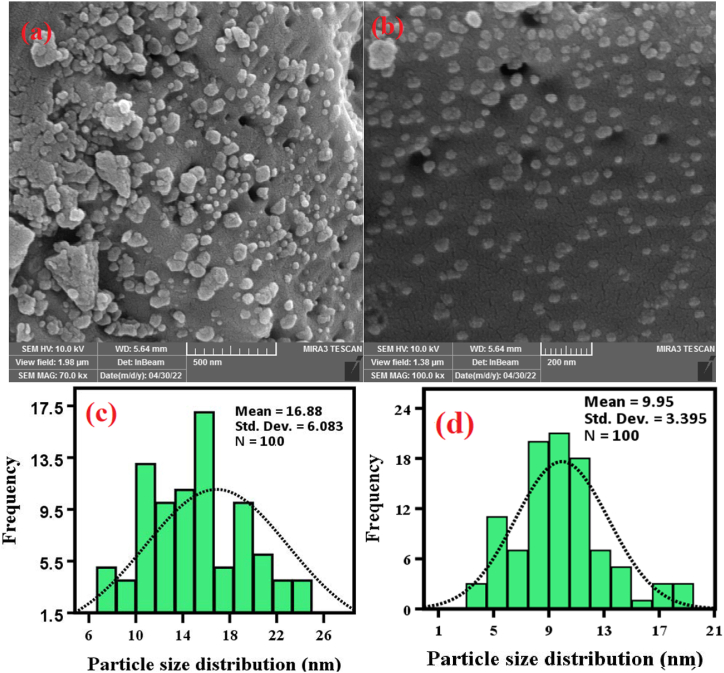
The FESEM images (a, b) and PSA (c, d) of NCP.

**Fig. 6:**
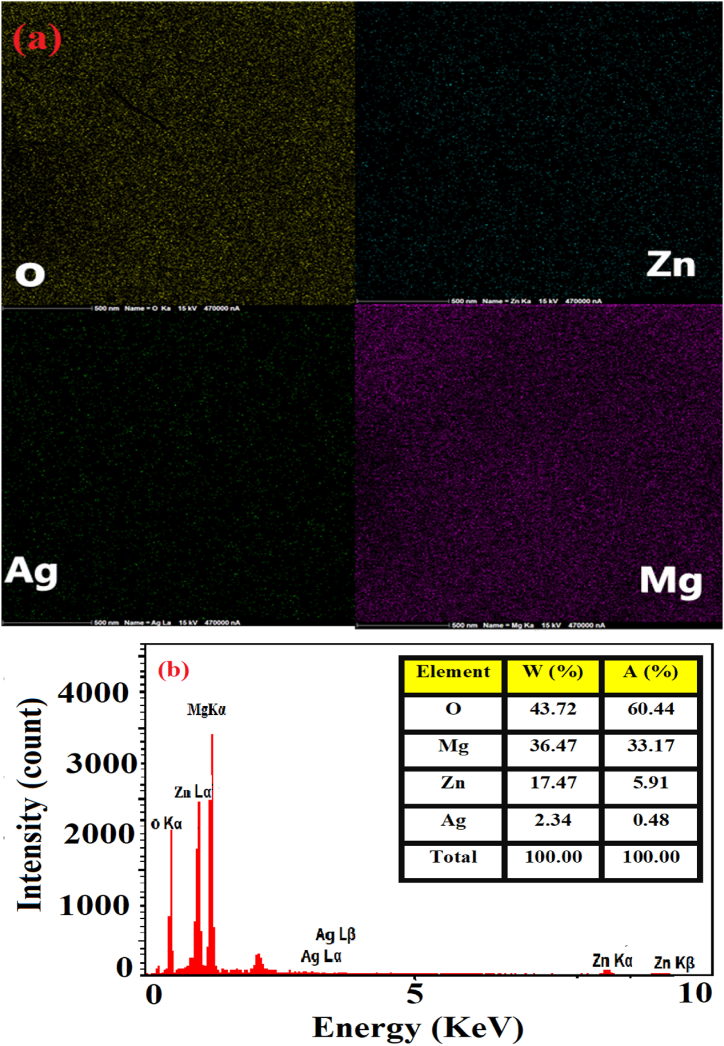
The mapping (a) and EDX (b) of Ag-doped ZnO/MgO-NCP..

The FESEM/PSA images of NCP (Ag-doped ZnO/MgO-NCPs, 600 °C) in Fig. 5 (a-d) indicate the formation of a spherical morphology with a size of about 9–16 nm. The EDX/Mapping images are presented in Fig. 6 (a, b). The analysis of EDX confirms the presence of Zn, Mg, Ag, and O elements and the absence of other impurities in the prepared nanocomposite, while the Mapping images display the uniform distribution of elements throughout the synthesized product.

### 3.5 Photocatalytic activity

#### 3.5.1 Photocatalytic process

**Fig. 7:**
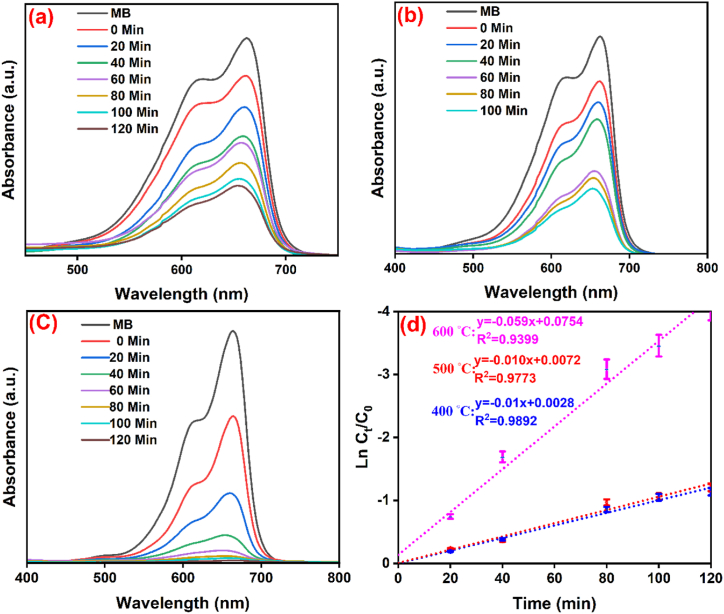
Degradation of MB (a = 400, b = 500, and c = 600 °C) by nanocomposite and the kinetic chart of MB (d).

**Fig. 8:**
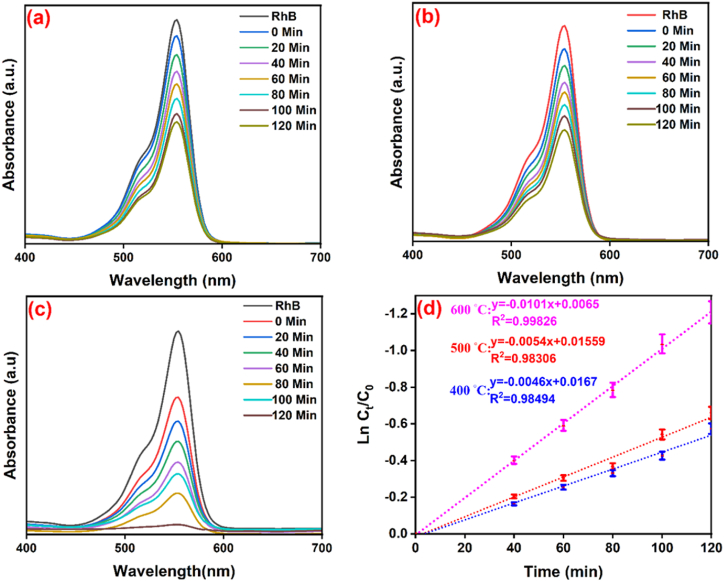
Degradation of RhB (a = 400, b = 500, and c = 600 °C) by nanocomposite and the kinetic chart of RhB (d).

The synthesized NCP at 600 °C, 500 °C, and 400 °C was investigated for photocatalytic process on the degradation of RhB and MB pigments in the presence of UV light, for which the MB and RhB solutions were significantly decolorized after 120 min. Fig. 7 (a-c, MB dye**)** and Fig. 8 (a-c, RhB dye**)** exhibit the degradation chart and specify the maximum wavelengths of MB ($\lambda_{\max}=663\;nm$) and RhB ($\lambda_{\max}=554\;nm$) in optimum conditions. Figs. 7 (d) and Fig. 8 (d) display the kinetic activity of MB and RhB, as the degradation of dyes was designated by applying Eq. (7) [42].

(7) $$\mathrm{Ln}\;\left(\frac{C_{t}}{C_{0}}\;\right)=K_{\text{obs}}$$

where C_0_ and C_t_ represent the concentrations of solution (t = 0) and concentrations of the solution that contained nanocomposite at irradiation time (t), individually [33]. In clarity, the rate constant (K_obs_) of photocatalytic degradation of nanocomposite/MB demonstrated better activity than the nanocomposite/RhB. The plots of Ln (C_t_/C_0_) against irradiation time are illustrated in Figs. 7 (d) and Fig. 8 (d). The results of MB and RhB degradation and rate constant (K_obs_) of synthesized nanocomposite at temperatures 400, 500, and 600 °C were shown in Table 2. Optimization parameters such as pH effect, amount of nanocomposite, and amount of MB have been investigated in the following.

**Table 2:** The results of MB and RhB degradation.

Temp (◦C)	Dye	Degradation (%)	K_obs_ (min^−1^)
400	MB	67	0.010
	RhB	58	0.0046
500	MB	70	0.010
	RhB	63	0.0054
600	MB	98	0.059
	RhB	97	0.0101

##### 3.5.1.1 Effect of pH

**Fig. 9:**
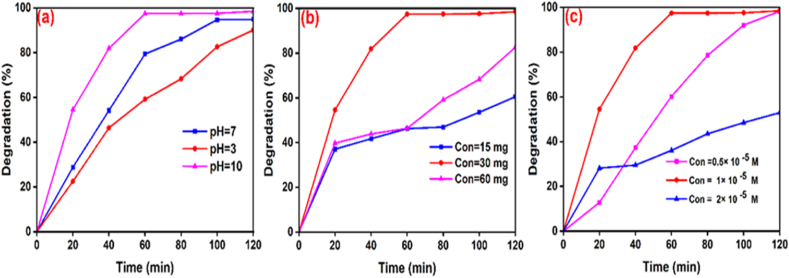
The effects of pH (a), the dose of nanocomposite (b), and MB amount (c).

In this research, the optimization of the pH parameter is estimated on the photocatalytic degradation of MB dye [43]. In this study, the concentration of MB dye (1 × 10^−5^ M) and photocatalyst dose (30 mg) was used in pHs 3, 7, and 9. Under UV-A light, degradation was done and absorption was read with a UV–Vis spectrophotometer. The amount of MB degradation was calculated through Eq. (1). According to the outcome of Fig. 9 (a), the highest degradation percentage was detected in an alkaline medium (pH = 9) about 98 %. MB is a cationic dye and since negatively charged particles are produced on the surface of the alkaline environment, it can do the destruction procedure faster [44].

##### 3.5.1.2 Amount of nanocomposite

To optimize the dose of the nanocomposite, MB solutions with a concentration of 1 × 10^−5^ M at pH = 9 were compared with different doses of the nanocomposite (15, 30, and 60 mg). The degradation percentage of MB was estimated by Eq. (1)**.** According to the result in Fig. 9 (b), the dose of 30 mg displayed the highest degradation percentage. An increase in the amount of nanocomposite causes to decrease in the degradation percentage of MB because the adding amounts of nanocomposite causes an accumulation of it [45].

##### 3.5.1.3 Amount of MB dye

To optimize the quantity of MB, solutions of nanocomposite with optimized concentration (30 mg) at pH = 9 were mixed with various doses of MB (0.5 × 10 ^−^^5^, 1 × 10 ^−^^5^, and 2 × 10 ^−^^5^ M). The degradation percentage of MB was calculated by Eq. (1). The results in Fig. 9(c) show an increase in the quantity of MB causes to decrease in the degradation percentage because the adding quantity of MB needs a longer time to get an adsorption equilibrium in the system [46].

#### 3.5.2 5. 2. photolysis activity

**Fig. 10:**
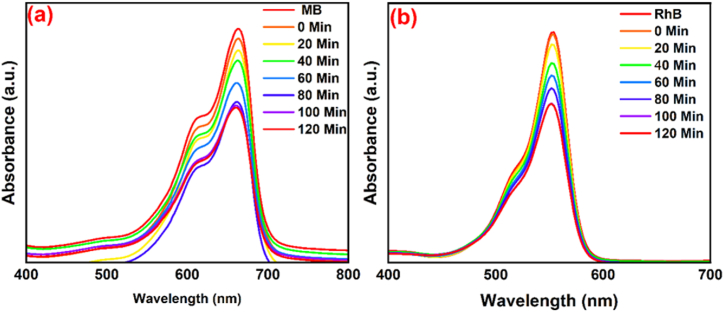
Photolysis activity for degradation of MB (a) and RhB (b) dyes.

The consequences of UV irradiation on the degradation of MB and RhB dyes were investigated in the lack of NCP; the obtained results are provided in Fig. 10 According to the results, the degradation percentage of MB and RhB dyes were about 33 % and 31 %, respectively.

**Fig. 11:**
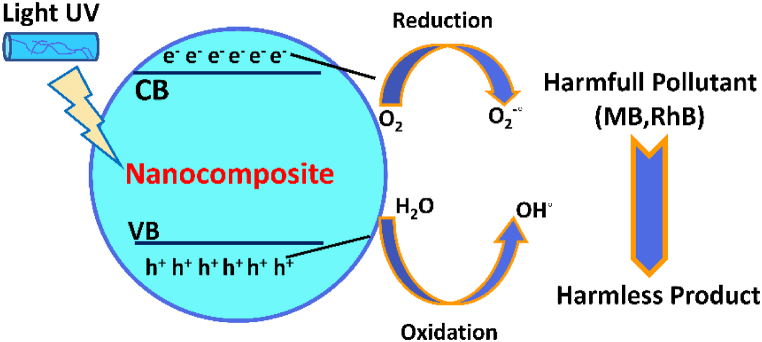
Mechanism of photodegradation of dyes.

The heterogeneous photocatalytic process begins when the amount of energy photons is tantamount or bigger than the band gap energy (Eg) of the nanocomposite, which causes the phenomenon of electron excitation. Initially, photo-generated holes $(h_{VB}^{+}$) and electrons ($e_{CB}^{-}$), are formed in the valence band and conduction band, respectively [33]. Then, the photocatalytic reaction (**Reactions 1–7**) facilitates the usage of these holes for the oxidation process to trap electrons for the reduction procedure [47]. The photodegradation mechanism of dyes using NPC is presented in Fig. 11 [[48], [49], [50], [51]]. The comparison of photocatalytic degradation of dyes with recent studies is presented in Table 3**.**

(8) $$Ag\;doped\;ZnO/MgO+h\nu \to \;\left(Ag\;doped\;ZnO/MgO\right)*\;\left(\;h_{VB}^{+}+e_{CB}^{-}\right)$$

(9) $$O_{2}+e_{CB}^{-}\to O_{2}^{•-}$$

(10) $$2H^{+}+2e_{CB}^{-}+O_{2}\;\to \;H_{2}O_{2\;}$$

(11) $$H_{2}+O_{2}^{•-}\;\to {OH}^{•}+O_{2}+{OH}^{-}$$

(12) $$MB+{OH}^{•}\to \;Degradation$$

(13) $$MB+O_{2}^{•}\to \;H_{2}O+{CO}_{2}\;\left(Oxidation\right)$$

(14) $$MB+h_{VB}^{+}\to \;H_{2}O+{CO}_{2}\;\left(Reduction\right)$$

**Table 3:** The comparison of photocatalytic degradation of dyes with recent studies.

Nanocomposite	Dye	Degradation (%)	Time (min)	*Ref.*
**ZnO–MgO**	RhB	83	90	[34]
**MgO/ZnO**	MB	89.5	120	[10]
**Ag-doped MgO**	MB	52 to 75	180	[52]
**Ag-doped ZnO/MgO**	MB	90	120	[53]
**Ag-doped ZnO**	MB	93	180	[54]
**ZnO**	RhB	95	180	[55]
**Ag/ZnO**	RhB	95	120	[56]
**Ag-doped ZnO–MgO–CaO**	MB	93	120	[30]
**Ag-doped ZnO/MgO**	MB	98	120	**This work**
	RhB	97		

### 3.6 Investigation of cytotoxicity

**Fig. 12:**
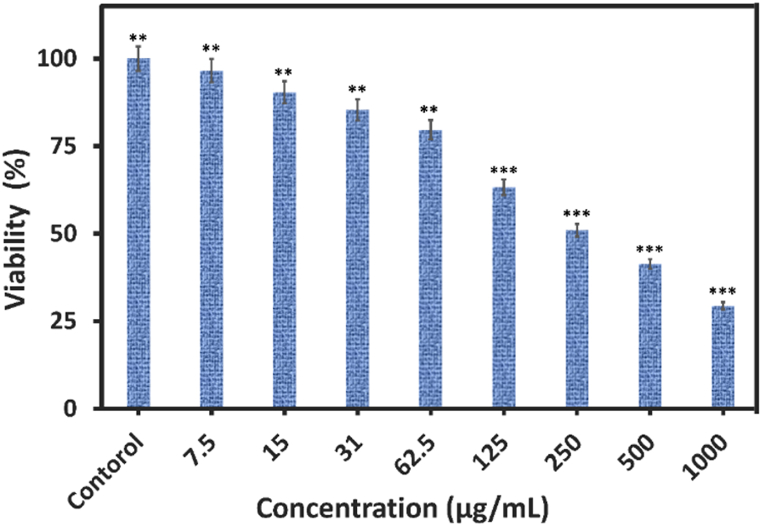
Evaluation cytotoxicity of NCP against cancer CT-26 cell line.

The cytotoxicity of our product in different concentrations was investigated on CT-26 cell lines (0–1000 μg/mL). Considering its concentration-depending toxicity, the nanocomposite caused significant toxic effects on the cancer cell lines after increasing the applied volume. The gathered outcomes are presented in Fig. 12 and according to the MTT assay, the nanocomposite induced the death of CT-26 cells after 24 h with the IC_50_ value of about 250 μg/mL. These observations confirm the effectiveness of this nanocomposite's cytotoxicity on CT-26 cancer cells as a drug carrier in cancer treatment.

## 4 Conclusion

In this study, Ag-doped ZnO/MgO-NCP was synthesized by utilizing *Mentha pulegium* plant extract. As the results of the XRD pattern confirmed its crystal structure, the FESEM/PSA images displayed spherical morphology with a size of about 9–16 nm. The presence of Zn, Mg, Ag, and O elements was proved by the EDX analysis. The results of photocatalytic activity at temperatures 400, 500, and 600 °C, showed the effectiveness of synthesized nanocomposite on the removal of MB dye in optimum conditions (pH = 9, nanocomposite amount ∼ 30 mg, and dye concentration ∼ 1 × 10^−5^ M) was about 98 % and also, the destruction percentage of RhB pigment was 97 % in pH = 9. The outcomes of MTT also confirmed the toxic effects of this product on the CT-26 cell line and approved its applicability as a drug supplement in cancer treatment.

## Funding

Not applicable.

## Ethical approval

For this type of study, ethical approval was not.

## Consent to participate

Not applicable.

## Consent for publication

Not applicable.

## Availability of data and material

Not applicable.

## CRediT authorship contribution statement

**Toktam Shekofteh Narm:** Writing – original draft, Investigation, Formal analysis, Data curation. **Habib Hamidinezhad:** Supervision, Project administration, Formal analysis, Data curation. **Zahra Sabouri:** Writing – review & editing, Investigation, Formal analysis, Data curation. **Majid Darroudi:** Writing – review & editing, Supervision, Project administration, Investigation, Funding acquisition.

## Declaration of competing interest

The authors declare that they have no known competing financial interests or personal relationships that could have appeared to influence the work reported in this paper.
